# Practical Aspects of Medical Science

**Published:** 1894-03-03

**Authors:** 


### Practical Aspects of Medical Science.

At a meeting the other day of the New York Medical Jurisprudence Society the subject of suicide and evolution was brought up for discussion. One theory which was offered in explanation of suicide is not without interest to the general practitioner, and, indeed, to the medical world in general. This is, that suicide is the outcome of evolution, resultant in the development of the higher centres at the expense of the lower, or instinct. Mr. Shortt, who offers this explanation, does not claim for it that it is more than a theory, but it is anyhow the result of much serious thought and consideration on his part. He points out to us that, perhaps, in no instance is there a deeper gulf fixed between the medical mind and the legal mind than in the way in which insanity is regarded respectively by both. Insanity is looked upon by the physician as merely an abnormal mental condition growing out of a set of physical facts, such as disordered nutrition or a diseased state of the nervous system. The lawyer, on the other hand, regards insanity from the aspect of morals or ethics. And the same difference exists between the view taken by the two professions with regard to suicide. It is regarded as a crime by the lawyer, to be punished as such, should the criminal fail in his purpose; whereas the physician considers it in the light of insanity due to an abnormal physical condition. He applies treatment in accordance with that view, disregarding the moral aspect of the question. If it were a determined question, Mr. Shortt asks, that suicide is a definite indication of insanity, would not the number of suicides then be in proportion to the number of the insane? In most countries the reverse of this is shown to be the case.

In England the rate per million inhabitants was, of the insane, 3,190; of suicides, 75; in Scotland, of insane, 3,215; of suicides, 35; or a slight increase of insane and marked decrease of suicides; in Ireland, of insane, 3,676; of suicides, 10; being a still greater increase of insane and decrease of suicides. Neither do suicide and insanity correspond in periods of occurrence. For instance, insanity increased at puberty, whereas suicide remained low between the tenth and twentieth year.

Statistics could again be appealed to in contrasting the prevalence of crime with that of suicide. Crime implied the presence of instinct in full force, yet the rate of suicides, Mr. Shortt pointed out, was the reverse of that of homicide. That is, the degree of civilisation where suicide was greatest was just the reverse of that where homicide prevailed. In the former, instinct was weaker or quiescent. In every portion of the world the extent of suicide corresponded with the extent of culture. In Denmark to-day, where education was carried furthest, the proportion of suicides was greater than in any other part of the world with the exception of France, or more particularly, Paris. In Arabia suicide is almost unknown. Culture is more widely distributed among men than among women; the latter were also more under the influence of instinct. Yet it has frequently been remarked upon that three men committed suicide to one woman. The growth of intelligence weakens all the instincts, Mr. Shortt urges, including also love of life, and it is this substitution of intelligent action for instinctive action which makes suicide possible.

A further and a practical evidence in favour of this exposition, that suicide is not an indication of insanity, but is influenced to some very appreciable extent by outside circumstances, was given by the author in the instance of the German Army. The component parts of this body are supposed by all to be a strong set of men, well disciplined and hardily brought up, and yet the number of suicides amongst them is enormous.

As bearing directly upon the question of evolution and suicide, the author further said that animals never committed suicide; he doubted it even in the instance of the scorpion, and in their case it was well known that instinct especially held sway. The lower the animal the more instinctive, he remarked, were its actions, intellect and volition not being particularly manifest until we come to man, in whom, were it not for instinct, there would be nothing to restrain from the act of suicide, even under minor degrees of provocation. The whole process of modern education and civilisation has a tendency to weaken instinct, our authority urges. It was under the predominance of intellect that occasions arose when a man might well question whether life was really worth living; whereas, in cases where instinct alone held sway, these introspections are not so frequent—man simply went on living.

We have most of us remarked, and it has frequently been pointed out to us, most noticeably, perhaps, by the guardians of the Morgue in Paris, that the pleasantest, brightest, and most hopeful months of the year are the ones selected by those who take their own life, whilst in the comparatively unpleasanter winter months of December and January the number of cases are at a minimum. Dr. Shortt's explanation of this is that there is a high state of activity during the severer months necessary to the prolongation of life, and a lesser strain during the warmer season. As soon as the successful effort relaxes, instinct thus also decreases. The further fact that suicides are generally at night time, points to the same conclusion on the part of our authority.

At present the student is only a thinking machine, says our American theorist, and a treatment to counteract the mental exercisement lies in such physical methods as would be most conducive of cultivating the instincts contemporaneously with the mental education. The animal, the instinctive nature, so to speak, is, according to Dr. Shortt, squeezed out of human nature in the latter-day process of civilisation. However much or however little one may be in sympathy with the view he takes of this question of suicide and evolution, one of the many vitally interesting ones of medical jurisprudence, we cannot let pass by unchallenged the assertion that the education of the day is confined to the development of the brain. In England this certainly is by no means the case; at our great Universities athletics are given a remarkably prominent place. A 'Varsity "blue" has a distinct commercial value of its own; whilst at our public schools almost the same necessity is attached to the physical as to the mental training of the youth of to-day.

